# Cannabis-related psychosis: Presentation and effect of abstinence

**DOI:** 10.4103/0019-5545.37665

**Published:** 2007

**Authors:** Vani Kulhalli, Mohan Isaac, Pratima Murthy

**Affiliations:** Department of Psychiatry, National Institute of Mental Health and Neurosciences, Hosur Road, Bangalore - 560 029, India; * Community, Culture and Mental Health Unit, School of Psychiatry and Clinical Neurosciences, University of Western Australia, Freemantle, WA 6160

**Keywords:** Cannabis, drug-induced psychoses, psychoses

## Abstract

**Background:**

The correlation between cannabis and negative mental health outcomes has been unequivocally established. Nevertheless, there is still a great need to research different dimensions of cannabis-related disorders, among which the study of cannabis-related psychosis is very important. There is a dearth of research regarding phenomenology and effect of abstinence, particularly from India. This study attempts to research the clinical presentation of cannabis-related psychosis and effect of abstinence.

**Aim:**

The aim of the present study was to document the clinical presentation of cannabis-related psychosis at presentation and after 7 days' abstinence from cannabis.

**Materials and Methods:**

Subjects with psychosis following cannabis use without any other prior or concurrent psychiatric disorder presenting to the outpatient department of a large tertiary care hospital were consecutively recruited for study. They were observed in a drug-free, protected environment for 7 days, during which clinical features were recorded using the Brief Psychiatric Rating Scale (BPRS).

**Results:**

Twenty male subjects were recruited and phenomenology was evaluated on the BPRS. Items with highest frequencies were unusual thought content (100%), excitement (75%), grandiosity (75%), hallucinatory behavior (70%) and uncooperativeness (65%). The least common symptoms were anxiety (5%), guilt feeling (5%), depressive mood (10%), motor retardation (10%) and blunted affect (30%). Nine subjects (45%) presented with cognitive dysfunction. Affective psychosis was the predominant diagnosis. At the end of 1 week of abstinence from cannabis, there was a significant decrease in scores. Significant improvement was observed in cognitive dysfunction, conceptual disorganization, grandiosity, tension, hostility, hallucinatory behavior and excitement.

**Conclusions:**

Cannabis-related psychosis presented with a predominantly affective psychosis and prominent thought disorder, excitement and violence. All subjects showed improvement in symptoms with abstinence from cannabis. A small heterogeneous sample and short duration of observation were the important limitations of this study.

One of the earliest reports about the effects of cannabis on mental health originated in India from the Indian Hemp Commission (1893).[1,2] Cannabis is widely used in India, which is an integral part of Indian culture and religious customs.[3,4] Despite of this, there is a dearth of research about cannabis from India. Among the studies are case reports, case series[3,5] and case-control studies[6] about cannabis-related psychoses.

Some studies have found cannabis-related psychosis to be a predominantly affective illness characterized by euphoria, increased psychomotor activity,[6–9] whereas others have found similarity to schizophrenia with prominent social withdrawal, thought disorder and disorganized behavior.[2,10–12] Some papers have not made this distinction.[2,13–17] There is a continuing need to define the presenting features of the disorder.[18]

Many papers have presented a one-time observation, often from records.[5,6] As a result, systematic recording was not possible. An observational study would overcome some of these difficulties. Extending the period of observation to a period of abstinence would add much to our data about the role of cannabis in psychosis. Only few researchers have studied the effects of abstinence from cannabis.[9,13]

This study aimed to document the presenting features of psychoses following cannabis use and the effect of 7 days of cannabis abstinence in a consecutive sample of 20 subjects from a large tertiary care hospital. The methodology was designed in a way to facilitate adequate observation and overcome some of the methodological deficits mentioned above.

## MATERIALS AND METHODS

The study was cleared by the Institute Ethics Committee. A series of 20 consecutive subjects presenting to the outpatient department and satisfying inclusion criteria were recruited for study after taking informed consent from subject and/or accompanying relative. The inclusion criteria were as follows:

- Any person over 16 years of age, who had psychosis and used cannabis at least three times a week for at least 1 month before onset of psychosis.[19]
- The most recent use of cannabis within a week prior to examination and presence of cannabis confirmed by testing the subject's urine for tetrahydrocannabinol (THC) using immunological card test (sensitivity 150 ng THC/mL of urine).

Any person with concurrent dependence on another substance in the 1 month preceding examination was excluded from the study. Due to feasibility considerations, persons using tobacco were included in the study. Subjects with history of mental illness prior to onset of cannabis use were excluded.

We selected subjects who had “psychosis following cannabis use”, which does not connote any causal relationship between cannabis and psychosis but only a temporal relationship as defined in the study.

During the study period, the subject was admitted in a protected (but not nicotine-free) environment, which ensured further abstinence from drug use. Acute behavioral disturbances were managed with PRN lorazepam. The study incorporated a week's period of observation. Subjects were not given any other psychotropic medication during this week. Every subject was assessed with a clinical interview, physical examination and baseline laboratory tests (hemogram, renal function, liver function and fasting blood sugar). Family history of mental illness in first-degree relatives was recorded. Information was elicited from both patient and a key informant. (Key informant was the one who knew the patient personally before illness onset and had witnessed the illness symptoms).

Subjects were assessed using the Brief Psychiatric Rating Scale (BPRS)[20] on the day of admission and then on second and seventh day thereafter (days 0, 2 and 7). BPRS is an 18-item, observer rated scale covering symptoms. It also has items to rate global psychopathology and change in psychopathology. The global improvement subscore changes were used to assess improvement as compared to baseline.

Statistical Package for Social Sciences (SPSS) was used for analysis, and significance level was at *P*-value less than 0.05.

## RESULTS

Twenty male subjects aged between 19 and 59 years (mean 30.15 years) were recruited for the study that was completed in 10 months.

An attempt was made to recruit every person who presented with the inclusion criteria to the outpatient services. However, nine persons had to be excluded (three were already on psychotropic medications, one refused inpatient admission, two left the hospital against the advise prior to completion of study and three in whom the information was inadequate).

### Cannabis use pattern

All subjects were cannabis smokers, and none used cannabis by other methods such as ingestion. The mean duration of cannabis use was 8.76 years (4 months to 25 years, SD 6.69 years). The quantity of cannabis use could not be estimated because of wide variation in potency of products used and unavailability of estimation techniques. All subjects were using cannabis almost everyday for at least 1 month prior to the onset of psychosis.

### Other substances used

The prevalence of lifetime use of alcohol was 95%, and 70% of the subjects had used it more than two times. Ninety-five percent of the subjects were concurrently using tobacco to the extent of dependence. Twenty-five percent of the subjects reported past history of using other drugs like opiates (10%), sedatives (10%) and volatile substances (5%). One subject had past history of benzodiazepine dependence. None of these substances was used to the extent of dependence in the month preceding examination as determined by history taking.

### Family history

The number of subjects having history of mental illness in at least one first-degree relative and their types are shown in Table 1. The commonest diagnosis is substance dependence disorder, predominantly alcohol.

**Table 1:** Subjects with family history positive for mental illness in at least one first-degree relative

Type of disorder	No. of subjects
Substance dependence disorder	9
Affective disorder	3
Nonaffective disorder	12
Other behavioral disorders	3
	

*Other behavioral disorder includes personality disorder and nonspecified disorders like somatization disorder, dementia and unspecified mental illness

### Baseline scores

The BPRS total scores ranged between 26 and 58 on the day of admission (day 0) with a mean of 41.6 and an SD of 8.96. Decrease in scores was reflected in serial scoring [Table 2]. The mean scores on days 0, 2 and 7 were 41.6, 33.9 and 31.55, respectively.

**Table 2:** Total Brief Psychiatric Rating Scale scores of subjects on day 0, day 2 and day 7 of study

BPRS scores	Minimum	Maximum	Mean	SD
Day 0	26	58	41.6	26
Day 2	18	54	33.9	10.4
Day 7	18	55	31.55	11.62

The highest number (by rank order) of subjects with positive scores on respective items of BPRS were [Table 5] unusual thought content (100%), excitement (75%), grandiosity (75%), hallucinatory behavior (70%) and uncooperativeness (65%). The least common symptoms were anxiety (5%), guilt feeling (5%), depressive mood (10%), motor retardation (10%) and blunted affect (30%).

**Table 5:** Change in symptom profiles (frequency of patients improved or worsened as per Global Improvement subscore of BPRS)

Item	Positive scores on day 0	Decrease in score on day 7	Increase in score on day 7
Somatic concern	4(20)	4(20)	1(5)
Anxiety	1(5)	1(5)	0
Emotional withdrawal	7(35)	5(25)	1(5)
Conceptual disorganization	10 (50)	7(35)	1(5)
Guilt feelings	1(5)	0	0
Tension	13 (65)	11 (55)	1(5)
Mannerisms and posturing	9(45)	8(40)	0
Grandiosity	15 (75)	10 (50)	2(10)
Depressive mood	2(10)	2(10)	0
Hostility	11 (55)	9(45)	2(10)
Suspiciousness	13 (65)	7(35)	3(15)
Hallucinatory behavior	14 (70)	10 (50)	1(5)
Motor retardation	2(10)	0	0
Uncooperativeness	13 (65)	8(40)	3(15)
Unusual thought content	20 (100)	10 (50)	3(15)
Blunted affect	6(30)	3(15)	3(15)
Excitement	15 (75)	12 (60)	3(15)
Disorientation	9(45)	6(30)	1(5)

Results indicate number of patients, Figures in parentheses are in percentage

### Other findings

Some useful findings of clinical examination are as follows: After discounting the history of cannabis use, 7 subjects qualified for a diagnosis of schizophrenia, 12 for a diagnosis of bipolar affective disorder mania with psychotic symptoms and 1 person for psychosis not otherwise specified (ICD-10). Eight subjects had first rank symptoms (Schneider). Delusions were commoner (65%) than hallucinations (45%) and types of delusions are as in Table 3. Eighty percent of the subjects had increased psychomotor activity. Other symptoms of psychoses such as disorganized behavior and catatonic symptoms (ICD 10[19]) were absent. No subject had syndromal depression while one patient was found to have obsessions and compulsions.

**Table 3:** Type of delusion in subjects

Type	No. of subjects
Grandeur	7
Persecution	5
Reference	4
Bizarre	4
Religious content	8

### Serial observation scores

There was a significant decrease in the total BPRS scores between days 0-2 and days 0-7 [Table 4]. The fall in scores was not significant in days 2-7. At study conclusion (day 7), maximum number of patients improved on excitement (60%), tension (55%), hostility (50%) and grandiosity (50%). Significant improvement occurred in items of conceptual disorganization, grandiosity, tension, hostility, hallucinatory behavior and excitement. Almost all the affected patients showed complete recovery in their cognitive functions by day 7 (as per global improvement subscore, one remaining patient improved in next 2 days. None of the patients was found to be asymptomatic at the end of the observation week. Some patients showed deterioration in specific symptoms [Table 5], but no subject showed worsening in total scores.

**Table 4:** Changes in the total BPRS scores in intervals of observation period. Student's t-test

Interval	t-value	df	P-value
Day 0 to day 2	3.471	19	0.003*
Day 2 to day 7	1.305	19	0.208 (NS)
Day 0 to day 7	3.944	19	0.001*

* Significant

## DISCUSSION

This study was aimed at determining the clinical symptoms in persons with cannabis use followed by psychosis. Therefore, we selected only those subjects having evidence of psychosis onset following regular use of cannabis and excluded the use of other mind-altering substances. The report of cannabis use was confirmed by urine testing for THC at admission into study.

The study showed a high comorbidity of substance abuse, especially alcohol and tobacco. A shared vulnerability to abuse a range of drugs has been suggested by some studies.[21–23] At our center, more than 90% of admissions are for alcohol abuse. We had to rely on the subjects' self-report of not using any other substances during the month before admission and were unable to objectively verify this report. Thus, the possibility that some symptoms were related to abstinence from other drugs cannot be completely excluded. This possibility was minimized by repeated clarification from subject and relative and checking surrogate indices of alcohol use (Gamma glutamyl transaminase levels). Patients were allowed for free access to tobacco; hence none of the symptoms may be attributed to abstinence from nicotine.

All subjects were male. This could be attributed to low levels of substance use among females in general and cannabis in particular.[24,25] All subjects were from low or middle socio-economic status families and had less than 10 years of formal education. This may reflect the general background of persons availing the hospital services. The age range was wide and may be due to variation in genetic vulnerability to psychosis, dose of cannabis and duration of illness, leading to variable age at presentation. This may also be viewed as a drawback of the study since homogeneity in duration of illness or episode number could not be ensured.

### Clinical picture at presentation

Forty-five percent of the subjects showed cognitive dysfunction in the form of disorientation in time and deficits in recent memory recorded by clinical history. Studies have reported occurrence of psychosis in clear sensorium[8] as well as with impaired cognitive function.[9–13] Cognitive dysfunction in these studies has varied from 100 to 30%. Both gross and subtle deficits have been reported.[14–17] Reports have also mentioned both temporary and permanent cognitive deficits in persons using cannabis. In our study, all subjects recovered from their cognitive deficits.

Maximum number of subjects scored on items of unusual thought content, excitement, grandiosity, hallucinatory behavior and uncooperativeness on the BPRS on the day of recruitment (day 0, Table 4). The high frequency of unusual thought content and hallucinatory behavior can be explained by the fact that these were among the criteria (psychotic symptoms[1]) for inclusion in the study. But other psychotic symptoms such as catatonic symptoms and disorganized behavior were absent. Positive symptoms (conceptual disorganization, grandiosity, suspiciousness, unusual thought content and hallucinatory behavior) were found at higher frequency than negative symptoms (emotional withdrawal, motor retardation and blunted affect). Most studies[26–29] have reported that cannabis produces psychosis with prominent positive symptoms. Our findings are in keeping with these reports. Although there was a mix of diagnosis in our sample, more numbers had an affective (manic) than nonaffective (schizophrenic) psychosis.

Sixty percent of the subjects were noted to have violent tendencies and attempted to assault staff and bystanders. In the BPRS, such behavior was reflected in positive scores on excitement and uncooperativeness. This finding is in keeping with previous reports of tendency to aggression and violence in cannabis-related psychoses.[8,18] Although anxiety and depression have been described as sequelae of cannabis use,[30] their role in the context of psychosis is not clear. In some studies, subjects were noted to be highly anxious and fearful due to overwhelming paranoid ideation.[16] In our study, very few subjects reported pervasive anxiety (5%) or depression (10%). Lorazepam may also have subdued anxiety in some subjects.

Scores on BPRS reflected both the total intensity and the profile of psychopathology; but it could not record, in detail, symptoms like delusions, obsessions and themes of psychopathology. However, these were recorded in the clinical interview. Maximum number of subjects scored positively on suspiciousness, grandiosity and unusual thought content while minority scored on somatic concern and guilt feelings. This tells us that a paranoid theme appeared in the psychopathology. Other studies have also described subjects as commonly having paranoid delusions.[3] As stated above, findings of clinical evaluation shed light on the themes and content of psychopathology. The mix of diagnoses replicates the findings of previous studies.[3,6,9,13] As in our study, previous authors have also alluded to different types of delusions, presence of first rank symptoms and religious and bizarre themes.[3,6,9,13]

Thus, our findings largely confirm reports of authors who have stated that cannabis produces a psychosis with predominantly affective features and more of positive symptoms, violence and excitement.

### Family history

The few studies that have examined family history in cannabis-related psychosis have reported that subjects gave a family history of substance dependence disorders, mental illness and personality difficulties.[6,13,26,29,31] In our study, it was noted that family history of substance abuse was most frequently reported. Family history of drug disorders is regarded as one of the risk factors for developing a drug dependence disorder, including cannabis dependence;[32] whether it also results in increased vulnerability to cannabis psychosis is not clear.

Subjects reported more of affective disorders in their family, suggesting a probable genetic vulnerability to affective symptoms. This may partly explain the predominance of affective psychosis in our sample. Some patients had a family history of more than one disorder. The information about family history was not analyzed in detail as it was not the focus of the study.

### Serial observation scores

All the subjects showed improvement during the week following admission, which was to a significant extent. This improvement occurred even in those subjects who did not receive lorazepam. The fact that all subjects improved with abstinence from cannabis without the use of any other psychotropic medication, indicates that cannabis may have either caused the psychosis or increased the intensity of psychosis. Most papers commenting on the outcome of cannabis-related psychoses have found that improvement occurs with abstinence.[6,9,16,31,33,34,38] Onyango[34] observed that cannabis use did not impact on psychosis in his sample. The findings of our study, however, clearly indicate that cannabis had a role in either causing or worsening but not in decreasing the symptoms of psychosis. Thus, we agree with the hypothesis that cannabis has a clear contributory role in producing psychosis in the user.

The observed improvement was noted for some specific symptoms. Maximum improvement was on items where the day 0 scores were high, so any change would have been easier to observe. On items with low scores on day 0, the changes may not have been so evident and therefore change was not noted. This may to some extent explain why some specific symptoms only improved.

Previous studies[6,9,16,31,33] have also noted improvement in specific symptoms. Rottanburg[9] had reported improvements in hypomania, agitation, self-neglect, delusions of persecution, delusion of reference, delusion of grandeur, irritability and sexual and fantastic delusions recorded serially by the present state examination (PSE). In our sample also, we noted improvement in similar symptoms. Several studies[27,33] have argued that cannabis produces worsening of positive symptoms. In our study also, we may note that specifically positive symptoms improved, while negative symptoms did not change much. The fact that some symptoms improved while others did not points to the possibility that cannabis has modified the picture of psychosis.

It can be argued then that this series may have included both toxic confusional states and cannabis psychosis as much of the improvement took place between day 0 and day 2. However, the primary aim of the study was not to examine this nosological issue but to study the effect of abstinence on psychopathology.

### Role of cannabis

There are questions about the “causal” role of cannabis in psychosis.[24,35–37] One way to examine the causal role is to recruit patients with cannabis use and observe the effects of abstinence, as done in our study. We could demonstrate temporal association and improvement with abstinence. To some extent, we could also demonstrate that cannabis produces a specific pattern of psychopathology. However, we were not able to demonstrate a dose-response relationship and the possibility that psychosis would not have occurred in absence of cannabis use. We may only infer that in our sample, cannabis contributed in either causing psychosis, increasing intensity of psychosis or in producing a particular combination of symptoms. After referring to the literature available, we hypothesized that cannabis contributes to produce psychosis, which has a distinct presentation. However, the generalization of our findings is limited by the small findings, inability to control for dose of cannabis use and genetic and other vulnerability to psychosis.

## CONCLUSIONS

Subjects in this study had high comorbidity with substance dependence, a predominantly affective (manic) psychosis with mental and physical restlessness, paranoid delusions, violent tendencies and improvement after cannabis abstinence. The implications are that cannabis contributes in causing and modifying psychosis and improvement is likely to occur with abstinence from cannabis. The important limitations of the study were small heterogeneous sample and a short duration of observation.

## References

[1] Negrete JC (1988). What's happened to the cannabis debate?. Br J Addict.

[2] Basu D, Malhotra A, Varma V (1994). Cannabis related psychiatric syndromes: A selective review. Indian J Psychiatry.

[3] Thacore VR (1973). Bhang psychosis. Br J Psychiatry.

[4] Suresh Kumar M (2002). Rapid assessment survey of Drug Abuse in India. Ministry of Social Justice and Empowerment and UN International Drug Control Programme, Regional Office of South Asia.

[5] Chopra GS, Smith JW (1974). Psychotic reactions following Cannabis Use in east Indians. Arch Gen Psychiatry.

[6] Basu D, Malhotra A, Varma V (1999). Cannabis psychosis and acute schizophrenia. Eur Addict Res.

[7] Thacore VR, Shukla SR (1976). Cannabis psychosis and paranoid schizophrenia. Arch Gen Psychiatry.

[8] Tunving K (1985). Psychiatric effects of cannabis use. Acta Psychiatr Scand.

[9] Rottanburg D, Robins AH, BenArie O, Teggin A, Elk R (1982). Cannabis associated psychosis with hypomanic features. Lancet.

[10] Harding T, Knight F (1973). Marijuana modified mania. Arch Gen Psychiatry.

[11] Tennant F S, Groesbeck J (1972). Psychiatric effects of hashish. Arch Gen Psychiatry.

[12] Channabasavanna SM, Paes M, Hall W, Hall W, Smart R (1999). Mental and behavioural disorders due to cannabis use in the book “The Health Effects of cannabis’.

[13] Carney P, Lipsedge M (1984). Psychosis after cannabis use. Br Med J.

[14] Hall W, Degenhardt L (2000). Cannabis use and psychosis: A review of clinical and epidemiological literature. Aust N Z J Psychiatry.

[15] Negrete JC (1973). Psychological adverse effects of cannabis-smoking: A tentative classification. C M A Journal.

[16] Talbott JA, Teague JW (1969). Marijuana psychosis, acute toxic psychosis associated with the use of cannabis derivatives. J Am Med Assoc.

[17] Thomas H (1993). Psychiatric symptoms in cannabis users. Br J Psychiatry.

[18] (1995). World Health Organisation.

[19] (1992). The ICD - 10 Classification of Mental and Behavioral Disorders.

[20] Overall JE, Gorham DR (1988). The brief psychiatric rating scale: Recent developments in ascertainment and scaling. Psychopharmacology.

[21] Tsuang MT, Lyons MJ, Meyer JM, Doyle T, Eisen SA, Goldberg J (1998). Co-occurrence of abuse of different drugs in men. Arch Gen Psychiatry.

[22] Fergusson DM, Horwood LJ (2000). Does cannabis use encourage other forms of illicit drug use?. Addiction.

[23] Degenhardt L, Hall W, Lynskey M (2001). Alcohol, cannabis and tobacco use among Australians: A comparison of their associations with other drug use and use disorders, affective and anxiety disorders and psychosis. Addiction.

[24] Stein MD, Cyr MG (1997). Women and substance abuse. Med Clin North Am.

[25] Jaffe JH, Kaplan, Kaplan (2000). Introduction and overview of substance related disorders. Comprehensive textbook of Psychiatry.

[26] Nunez LA, Gurpegui M (2002). Cannabis induced psychosis: A cross sectional comparison with acute schizophrenia. Acta Psychiatr Scand.

[27] Sarabjit Singh Mendhiratta, Wig NN, Verma SK (1978). Some psychological correlates of long term heavy cannabis users. Br J Psychiatry.

[28] Deahl M (1991). Cannabis and memory loss. Br J Addict.

[29] Ashton CH (2001). Pharmacology and effects of cannabis: A brief review. Br J Psychiatry.

[30] Palsson A, Thulin SO, Tunving K (1982). Cannabis psychosis in South Sweden. Acta Psychiatr Scand.

[31] Drummond L (1982). Cannabis psychosis: A case report. Br J Addict.

[32] Peralta V, Cuesta MJ (1992). Influence of cannabis use on schizophrenic psychopathology. Acta Psychiatr Scand.

[33] Andreasson S, Allebeck P, Rydberg U (1989). Schizophrenia in users and nonusers of cannabis. Acta Psychiatr Scand.

[34] Patton GC, Coffey C, Carlin JB, Degenhardt L, Lynsky M, Hall W (2002). Cannabis use and mental health in young people: Cohort study. Br Med J.

[35] Carney MW, Bacelle L (1984). Psychosis after cannabis abuse. Br Med J.

[36] Merikangas KR, Stolar M, Stevens DE, Goulet J, Preisig MA, Fenton B (1998). Familial transmission of substance abuse disorders. Arch Gen Psychiatry.

[37] Kolansky H, Moore WT (1971). Effects of marijuana on adolescents and young adults. J Am Med Assoc.

[38] Linzen DH, Dingemans PM, Lenior ME (1994). Cannabis abuse and the course of recent onset schizophrenic disorders. Arch Gen Psychiatry.

